# Understanding Libertarian Morality: The Psychological Dispositions of Self-Identified Libertarians

**DOI:** 10.1371/journal.pone.0042366

**Published:** 2012-08-21

**Authors:** Ravi Iyer, Spassena Koleva, Jesse Graham, Peter Ditto, Jonathan Haidt

**Affiliations:** 1 Department of Psychology, University of Southern California, Los Angeles, California, United States of America; 2 Department of Psychology and Social Behavior, Irvine, California, United States of America; 3 Department of Psychology, Charlottesville, Virginia, United States of America; Boston College, United States of America

## Abstract

Libertarians are an increasingly prominent ideological group in U.S. politics, yet they have been largely unstudied. Across 16 measures in a large web-based sample that included 11,994 self-identified libertarians, we sought to understand the moral and psychological characteristics of self-described libertarians. Based on an intuitionist view of moral judgment, we focused on the underlying affective and cognitive dispositions that accompany this unique worldview. Compared to self-identified liberals and conservatives, libertarians showed 1) stronger endorsement of individual liberty as their foremost guiding principle, and weaker endorsement of all other moral principles; 2) a relatively cerebral as opposed to emotional cognitive style; and 3) lower interdependence and social relatedness. As predicted by intuitionist theories concerning the origins of moral reasoning, libertarian values showed convergent relationships with libertarian emotional dispositions and social preferences. Our findings add to a growing recognition of the role of personality differences in the organization of political attitudes.

## Introduction

*“Civilization is the process of setting man free from men.”*

- *Ayn Rand (1944)*

Political psychologists have learned a great deal about the psychological differences between liberals and conservatives [1]–[4], but very little is known about the psychological characteristics of libertarians, who are sometimes described as being conservative on economic issues (e.g., against government regulation of free markets) but liberal on social issues (e.g., against government intrusion into private matters like sex or drug use). In the United States, libertarians appear to be rising in both numbers [5] and prominence in national politics [6]. The presidential candidacies of Texas Congressman Ron Paul in 2008 and 2012 and the 2009 birth of the “Tea Party” movement have greatly elevated the visibility and importance of libertarian ideas about individual liberty and the importance of limited government. Many “Tea Party” members are actually socially conservative [7], but emphasize ideas about limited government that reflect libertarian principles. In this paper, we document libertarian moral psychology, which, as we show, is distinct from both liberal and conservative moralities. We use this unique group to illustrate how psychological dispositions predispose individuals to endorse particular values and choose coherent ideological identifications, consistent with current models of moral intuitionism [8], ideological choice [9], and the moralization of preferences [10].

### Beyond the Bipolar View of Political Personality

The “culture war” fought out in American public and political life since the 1980s has often been described in binary terms as a conflict between two visions of morality and moral authority [11], [12]. On the right, the conservative side has insisted that there is an objective moral truth. Traditional institutions are seen as embodying the wisdom of the ages, and therefore closely reflecting this moral truth. On the left, the liberal side has insisted that moral truth is not fixed for all time, but is a work in progress, to be reinterpreted toward the goal of promoting greater well-being for all [11]–[13]. Psychologists have been able to measure these differences in moral judgment [3] along with their underlying personality correlates. For example, political conservativism has been found to be associated with greater tolerance of inequality, and lesser tolerance of change [4], greater conscientiousness [1], and greater sensitivity to disgust [14]. Political liberals, on the other hand, tend to be more open than conservatives to new experiences [1] and more empathic [15]. This research has been an important first step in understanding the ideology-personality relation, and the psychological organization of political attitudes. Rozin [16] highlighted the importance of identifying real-world invariance in meaningful parts of life, for which political ideology certainly qualifies.

Yet within research on ideology, libertarianism—with its mix of liberal and conservative sensibilities—has gone largely unstudied. Libertarian ideology prescribes a unique pattern of moral concerns that cannot be readily classified on the standard left-right dimension, but as with differences between liberals and conservatives, these unique sensibilities should be measurable using existing psychological scales. In this paper, we empirically address the question of what dispositional traits, emotions, and social preferences predict self-identification as libertarian. Based on the stated beliefs of libertarian intellectual leaders, as well as previous research on the social and intuitive origins of moral beliefs [17], we generate three broad expectations about libertarian psychology and evaluate them in a large dataset, across a variety of psychological characteristics. In addition to providing a detailed empirical description of the distinct moral-psychological profile of individuals who self-identify as libertarians in the US context, we examine the relations between their dispositional traits, values, and social preferences allowing us more general insight into the origins of moral judgment, which can then be applied to any group with this distinct psychological profile.

### Libertarian Ideology

Modern libertarians are attitudinally diverse, but all types of libertarianism trace their origins back to the enlightenment thinkers of the 17^th^ and 18^th^ century who argued that states, laws, and governments exist for the benefit of the people. The *individual* is the unit of value, and the liberty of the individual is the essential precondition for human flourishing. John Locke wrote: “the great and chief end, therefore, of men's uniting into commonwealths and putting themselves under government is the preservation of their property” ([18] - Para 123). Locke had an expansive notion of property, which included men's “lives, liberties, and estates.” His ideas were later paraphrased into one of the most famous phrases in the Declaration of Independence: “life, liberty, and the pursuit of happiness.”

Libertarianism has historically rejected the idea that the needs of one person impose a moral duty upon others. This is one of the major points on which liberals and libertarians diverged in the 20^th^ century. Libertarianism stayed close to Locke's and Mill's notions of liberty as freedom from interference, which the philosopher Isaiah Berlin [19] later called “negative liberty.” But beginning in the progressive era of the late 19^th^ century, the American left began to adopt European ideas about the conditions and entitlements that people need to make the most of their liberty. Government action came to be seen as essential for ensuring “positive liberty” by providing the social conditions – such as education, health care, and financial security – that give people the freedom to pursue their own happiness.

Seen in this light it becomes clear why American libertarians are sometimes called “classical liberals,” and in Europe, the term liberal is often used in the same way that “classical liberal” is used in the United States. It also becomes clear why libertarian thought is now associated with anti-government and anti-progressive movements. Libertarianism provides an ideological narrative whereby the opposition to high taxes and big government is not just an “economic” position: it is a *moral* position as well. This narrative provides the basis for principled opposition to a government seen as unfair (because it takes from the productive and gives to the unproductive), tyrannical (because it violates the negative liberty of some people to promote the positive liberty of others), and wasteful (because governments rarely achieve the efficiencies generated by the competition of private firms).

### The Psychological Roots of the Libertarian Ideology

The most obvious psychological characteristic of libertarian ideology is the value placed on negative liberty as an overriding moral principle, as can be seen in this quote concerning a law outlawing online gambling, from U.S. Congressman Ron Paul [20], the most libertarian contender in recent times for the nomination of a major political party:

The most basic principle to being a free American is the notion that we as individuals are responsible for our own lives and decisions. We do not have the right to rob our neighbors to make up for our mistakes, neither does our neighbor have any right to tell us how to live, so long as we aren't infringing on their rights…. There are those that feel online gambling is morally wrong and financially irresponsible, which I do not argue with, but they also feel that because of this, the government should step in and prevent or punish people for taking part in these activities. This attitude is anathema to the ideas of liberty.

Libertarians appear to have a coherent moral philosophy, which includes a general opposition to forcing any particular moral code upon others. Note that Paul is not saying that gambling is morally acceptable. Rather, he is saying that (negative) liberty has a moral value that supersedes other moral considerations. Libertarians seem willing to reject both liberal concerns for social justice [21] and conservative concerns for respecting existing social structure [22] when those concerns conflict with their superordinate interest in maintaining individual liberty. The goal of our first study is to confirm these observations by directly surveying a broad range of moral values and concerns, and testing whether self-described libertarians place a higher value on liberty and a lower value on other moral concerns, compared to self-described liberals and conservatives.

But what might explain the libertarian focus on liberty to the exclusion of other moral concerns? Recent work in moral psychology suggests that moral attitudes arise, at least in part, from low-level “dispositional traits” [23], emotional reactions [8], [24], social function [17], and the moralization of preferences [10]. These moral attitudes have, in turn, been found to be associated with ideological self-identification [3], [9].

This work suggests that one explanation for the unique moral profile of libertarians is that they *feel* traditional moral concerns less than do most other people. Tetlock, et al. [25] found that libertarians were less morally outraged by “taboo” moral tradeoffs (e.g., buying and selling body parts for transplantation) than were liberals, conservatives, or socialists. Recent research in moral psychology has emphasized the importance of intuitive and emotional reactions in producing moral judgments that appear, on their face, to be based on principled reasoning [8], [24], [26]. Might libertarians be more tolerant on issues of private consensual behavior than conservatives because they exhibit lower levels of disgust sensitivity [27]? Might libertarians depart from liberals on social justice issues because they have weaker feelings of empathy [15]? Indeed, libertarian writers have historically been proud of the rational — rather than emotional — roots of their ideology [28]. The possible exception to this rule, of course, is the vigorous reaction libertarians often have to violations of personal freedom. Libertarians' characteristic pattern of emotional reactions (and lack thereof) may constrain the types of concerns that they moralize, which in turn affects their attraction to libertarian self-identification. We investigate this possibility in Study 2.

Finally, emotional reactions, and the moral principles that derive from them, serve interpersonal functions [17], [29], such as navigating the social world [30] and forming groups with others [31]. Libertarians may have a dispositional preference for independence, perhaps even for solitude, and therefore less use for moral principles that bind them to others. In *The Fountainhead*, Ayn Rand [32] writes about the importance of maintaining one's individuality within social relationships. Do libertarians identify less with the people in their lives, with groups, and with their nations? Do they derive less enjoyment from the company of others? This relative preference for individualism may gradually become moralized into a conscious endorsement of liberty as a moral principle [10], predisposing them to a libertarian self-identification. We investigate these possibilities in Study 3.

### The Current Research

In this paper, we let libertarians speak for themselves. We report the results of 16 surveys in which a total of 11,994 self-identified libertarians participated. We show how self-described libertarians differ from self-described liberals and conservatives not just on their moral beliefs, but on a variety of personality measures that, given previous research on the emotional [8], [30] and social origins of moral reasoning [17], [29], [33], help us to understand *why* libertarians may hold their unique pattern of moral beliefs.

Our goal, however, was not just to describe the moral intuitions and dispositional traits of libertarians. Our second goal was to provide further evidence for the dispositional origins of ideology [1], [9], the role of intuition in moral attitudes [8], and the role that social functioning plays in moral thinking [17], [29], [33]. More specifically, we sought to replicate tests of a predictive model of ideological identification [9] that is similar to McAdams' framework of personality. McAdams' [34], [35] three-level account of personality posits that the lowest level consists of global, decontextualized “dispositional traits,” such as the Big 5 or disgust sensitivity. Level 2 refers to a person's “characteristic adaptations” such as values, goals attachment styles, and defense mechanisms. McAdams' third level consists of “integrative life stories,” which are the idiosyncratic stories that people tell themselves about themselves. These stories often weave the level 1 and level 2 constructs into narratives that help people understand and justify their particular moral values. Haidt, Graham, and Joseph [36] modified McAdams' third level for work in political psychology by pointing out that not all of these stories are self-constructed. We do not explicitly examine integrative narratives in this study, but when one gravitates toward an existing political party or ideology, one takes on many of the ideological narratives that have been laboriously constructed over decades by authors such as Ayn Rand (who, not coincidentally, put most of her political philosophy into narrative form in her novels).

To apply this model to the study of libertarians, we first show that libertarians do indeed have a distinct profile of moral concerns (Study 1). We then show that dispositional traits relate to ideological identification, and that this relationship is often mediated by moral intuitions, which can be thought of as a type of characteristic adaptation in McAdams' terminology (Study 2). In Study 3, we show that specific moral concerns relate to distinct styles of social functioning, and that libertarians' unique moral profile relates to their social preferences. Consistent with theories of parallel constraint satisfaction [37], we show that libertarianism can be understood as a set of relationships between a broad number of dispositional traits, social preferences, and moral values.

We begin with three general predictions.

1. *Libertarians will value liberty more strongly and consistently than liberals or conservatives, at the expense of other moral concerns.* This expectation is based on the explicit writings of libertarian authors (e.g. the Libertarian party website at lp.org, with the title “The Party of Principle: Minimum Government, Maximum Freedom”).
2. *Libertarians will rely upon emotion less – and reason more – than will either liberals or conservatives*. This expectation is based upon previous research on the affective origins of moral judgment [8], as well as libertarians' own self-characterizations. For example, one of the main libertarian magazines is called, simply, *Reason*.
3. *Libertarians will be more individualistic and less collectivist compared to both liberals and conservatives*. This expectation is based upon previous research concerning the social function of moral judgment [17], [29], [33]. Libertarians often refer to the “right to be left alone” [38], and show strong reactance toward social or legal pressures to join groups or assume obligations toward others that are not freely chosen [39].

We evaluate these predictions in three studies using large web-based samples and a variety of measures related to morality, cognition, emotion, and social relatedness. Each “study” is actually a collection of separate studies that were conducted via a data collection website (described below), but for presentation purposes, we group them together based on the predictions they address.

## Methods

### Participants and Sampling Considerations

The analyses presented are based on data from 157,804 participants (45.6% female, median age = 34) who visited YourMorals.org and participated in one or more studies between June 2007 and January 2011. Results replicate within sub-samples collected before and after January 2010, indicating that the findings of this paper were not greatly affected by current events that occurred during data collection. Only participants who were raised in the United States until at least the age of 14 were included in these analyses. YourMorals.org is a data collection platform where, after providing basic demographics, participants are invited to take part in any of 6–8 featured and 30–40 overall studies, each described with a title and a brief one sentence description. Before each study, participants were presented with an IRB approved information sheet, detailing our contact information, participant rights, and study details, to which they were asked to agree. Upon completion of each scale, a graph including the participant's own score in comparison to others is provided. Participants usually find YourMorals.org through publicity about psychological research or by typing keywords related to morality into a search engine. Most participants took one or two surveys, but 37% completed more than two, and 15% completed more than five.

YourMorals.org offers a unique opportunity to examine libertarian morality because, unlike most major surveys (e.g., Gallup, ANES), it allows participants to choose the label “libertarian” as a self-descriptor, rather than forcing them to select a point on the liberal-conservative spectrum. As of January 2011, 11,994 American visitors to YourMorals.org had self-identified as “libertarian”. Because our sample is not representative, we do not claim to describe the absolute percentage of libertarians who hold any particular belief or share any particular trait. Rather, our goal is to compare libertarians to liberals and conservatives on a variety of personality traits, in order to examine whether the relationships found between dispositions, values, and social functioning in previous research are also found within self-described libertarians.

### Overall Design

Our main dependent variable is political self-identification, which we use to compare ideological groups within each specific study. Upon registration, participants were asked “When it comes to politics, do you usually think of yourself as liberal, moderate, conservative, or something else?” Options available on a dropdown menu included “very liberal,” “liberal,” “slightly liberal,” “moderate/middle of the road,” “slightly conservative,” “conservative,” “very conservative,” “don't know/not political”, “libertarian,” and “other.” The libertarian option was chosen by 7.6% (N = 11,994) of American visitors; 13.5% (N = 21,278) chose one of the three conservative options, 11.1% (N = 17,541) chose “moderate,” while the majority (61.5%, N = 97,021) chose one of the three liberal options.

Examining libertarians' responses on social versus economic issues indicated that our participants' understanding of the term “libertarian” converged with our expectations that they would be fiscally conservative and socially liberal. When asked “how liberal or conservative” they were on “economic issues” on a 1 (very liberal) to 7 (very conservative) scale, libertarians indicated that they were even more conservative (M = 6.10) than conservatives (M = 5.93), and far more conservative than liberals (M = 2.83). When asked the same question about “social issues”, libertarians characterized themselves as much more liberal than self-identified conservatives (M's = 2.49 vs. 5.16), though not as liberal as liberals themselves (M = 1.66). Self-identified libertarians in our sample also reported specific political attitudes that were consistent with the expected pattern of relative social liberalism. For example, 59% of libertarians felt that “abortion should be generally available to those who want it” compared with 18% of conservatives, and 69% of libertarians felt “same sex couples should be allowed to legally marry” compared with 21% of conservatives (the comparable percentages for self-identified liberals were 84% for abortion and 92% for gay marriage). Parallel analyses using social liberalism combined with fiscal conservatism as a proxy for libertarianism in this sample replicated the main findings of this paper. Analysis of the values of a statistically extracted cluster (see Table 1) also replicates the general pattern found using self-identification. However, we report results for those who self-categorized as libertarian, as we believe that self-categorization is a significant psychological step that corresponds to how libertarianism is used in American political discourse (e.g. the libertarian party is an active third party).

**Table 1:** Description of Three Groups from Study 1 Cluster Analysis.

	Group 1	Group 2	Group 3
Number in Group	2003	737	354
MFQ-Harm mean	3.65	3.26	2.21
MFQ-Fairness mean	3.67	3.20	2.85
MFQ-Ingroup mean	2.09	3.15	1.70
MFQ-Authority mean	2.01	3.35	1.56
MFQ-Purity mean	1.32	3.15	0.65
MFQ-Lifestyle Liberty mean	3.95	3.41	4.67
MFQ-Economic Liberty mean	2.44	3.63	4.24
% Liberal	73.6	17.5	17.5
% Conservative	3.8	44.1	5.6
% Libertarian	6.1	11.7	59.6
% Moderate	8.5	17.2	4.8
% Other/Don't Know/Apolitical	8.0	9.5	12.4

The specific sub-sample that elected to take each study is described along with each measure. The full libertarian sample was mostly white (87.5% of those who answered our ethnicity question), male (79.6%), well educated (79.3% were in college or had earned a college degree), and diverse on age (mean age = 34.88, SD = 13.1). Libertarians were comparable to other participants in terms of education, ethnicity, and age, but were much more likely to be male (79.6%) compared to both liberals (50.6% male) and conservatives (63.0% male). Because of this difference and because many of the distinguishing characteristics of libertarians turn out to be traits on which there are substantial gender differences, we include tables that show the effects separately for males and females.

Due to our large sample sizes and the many differences between liberals and conservatives, virtually all measures produced highly significant contrasts. Because the degree of significance is not as important as the overall pattern of differences, we do not discuss *p* values in the text, though they are indicated in Tables 2, 3, and 4. Differences are shown as Cohen's *d* scores in these tables to allow comparisons of effect sizes across scales and genders. Any time we say that one group scored higher or lower than another group, the difference was significant at *p*<.01, and usually at *p*<.001. In describing our effects, we generally follow Cohen's [40] classification of effect sizes as small/slightly (*d* = .10 to .39), medium/moderately (*d* = .40 to .69), or large/substantially (*d*>.70), but give the exact *d* statistic when differences are very small or very large. Rather than describing each measure in a single method section, we provide a short description of each scale and its sample, followed by the results for that scale, and a brief discussion of how the results help us evaluate our three predictions. Finally, after each set of measures, many of which have a great deal of psychological overlap, we include multivariate analyses designed to help the reader synthesize each set of measures presented.

**Table 2:** Means and Cohen's d-scores for measures in Study 1.

	Means			Cohen's d-score for Libertarians Compared to Liberals						Cohen's d-score for Libertarians Compared to Conservatives					
Scale	Libertarians	Liberals	Conservatives	Overall		Men		Women		Overall		Men		Women	
**Moral Foundations Questionnaire**															
Harm	2.73	3.68	3.03	−1.15	**	−1.07	**	−0.94	**	−0.34	**	−0.23	**	−0.35	**
Fairness	3.09	3.76	3.02	−0.96	**	−0.95	**	−0.87	**	0.08	**	0.13	**	0.06	
Ingroup	2.25	2.14	3.12	0.13	**	0.10	**	0.16	**	−1.05	**	−1.07	**	−1.03	**
Authority	2.16	2.12	3.32	0.06	**	0.04	*	0.11	**	−1.42	**	−1.44	**	−1.35	**
Purity	1.35	1.37	3.00	−0.02		0.02		0.06		−1.57	**	−1.51	**	−1.61	**
**Schwartz Values Scale**															
Achievement	4.35	4.25	4.37	0.10	*	0.14	**	0.00		−0.02		−0.01		−0.08	
Benevolence	4.01	4.65	4.53	−0.62	**	−0.51	**	−0.63	**	−0.50	**	−0.44	**	−0.54	**
Conformity	2.86	2.96	4.18	−0.08		−0.03		−0.11		−1.04	**	−1.01	**	−1.09	**
Hedonism	3.97	3.81	3.14	0.11	**	0.06		0.12		0.55	**	0.60	**	0.43	**
Power	1.85	1.78	2.29	0.06		0.04		0.02		−0.34	**	−0.34	**	−0.37	**
Security	3.52	3.60	4.30	−0.08		−0.01		−0.08		−0.79	**	−0.75	**	−0.83	**
Self-Direction	5.36	5.13	4.79	0.27	**	0.29	**	0.24	**	0.61	**	0.61	**	0.61	**
Stimulation	3.39	3.42	2.83	−0.02		−0.06		0.00		0.34	**	0.33	**	0.37	**
Tradition	1.73	1.93	3.23	−0.16	**	−0.13	**	−0.21	**	−1.15	**	−1.12	**	−1.23	**
Universalism	3.65	4.84	3.51	−1.06	**	−1.03	**	−0.88	**	0.12	**	0.13	*	0.26	*
**Ethics Position Questionnaire**															
Idealism	2.78	3.29	2.96	−0.64	**	−0.53	**	−0.62	**	−0.21	**	−0.07		−0.38	**
Relativism	3.06	3.27	2.49	−0.25	**	−0.27	**	−0.09		0.58	**	0.61	**	0.63	**
**Adapted Good Self Scale**															
Moral Traits	2.84	3.24	3.28	−0.73	**	−0.71	**	−0.60		−0.76	**	−0.61	*	−1.07	**
Pragmatic Traits	3.04	3.03	3.07	0.02		0.00		0.20		−0.06		0.00		−0.10	
**Liberty Foundation**															
Lifestyle Liberty	4.47	3.89	3.51	0.81	**	0.80	**	0.72	**	1.19	**	1.16	**	1.16	**
Economic Liberty	4.27	2.32	3.88	2.56	**	2.65	**	2.40	**	0.52	**	0.45	**	0.57	**

*Note:*

* *p<.01,*

** *p<.001 (two tailed).*

**Table 3:** Means and Cohen's d-scores for scales in Study 2.

	Means			Cohen's d-score for Libertarians Compared to Liberals						Cohen's d-score for Libertarians Compared to Conservatives					
Scale	Libertarians	Liberals	Conservatives	Overall		Men		Women		Overall		Men		Women	
Big Five Personality Inventory															
Agreeableness	3.36	3.64	3.60	−0.45	**	−0.38	**	−0.44	**	−0.37	**	−0.27	**	−0.46	**
Conscientiousness	3.39	3.47	3.62	−0.11	**	−0.01		−0.04		−0.33	**	−0.29	**	−0.29	**
Extraversion	2.96	3.12	3.10	−0.19	**	−0.09	**	−0.17	**	−0.16	**	−0.11	**	−0.17	**
Neuroticism	2.69	2.88	2.70	−0.23	**	−0.21	**	0.00		−0.01		0.01		0.16	**
Openness	4.06	4.08	3.75	−0.04		−0.15	**	0.07		0.50	**	0.41	**	0.63	**
Interpersonal Reactivity Index															
Empathic Concern	3.21	3.92	3.57	−0.91	**	−0.81	**	−0.76	**	−0.44	**	−0.33	**	−0.42	**
Fantasy	3.51	3.75	3.46	−0.28	**	−0.22	**	−0.09		0.06		0.05		0.26	*
Personal Distress	2.14	2.41	2.23	−0.36	**	−0.32	**	−0.18		−0.11		−0.04		−0.06	
Perspective Taking	3.52	3.69	3.40	−0.24	**	−0.27	**	−0.09		0.15	*	0.17	*	0.23	*
Disgust Scale	1.52	1.63	1.91	−0.20	**	−0.07	*	−0.03		−0.61	**	−0.49	**	−0.68	**
Hong Reactance Scale	3.40	3.15	3.01	0.43	**	0.49	**	0.29	*	0.65	**	0.69	**	0.54	**
Baron-Cohen															
Empathizer	2.71	3.04	2.88	−0.76	**	−0.60	**	−0.65	**	−0.38	**	−0.34	**	−0.32	*
Systemizer	2.89	2.67	2.76	0.49	**	0.22	**	0.41	**	0.31	**	0.15	*	0.42	**
Need for Cognition	4.24	4.15	3.93	0.17	**	0.11		0.13		0.54	**	0.49	**	0.61	**
Moral Dilemma - Utilitarianism															
Overall	−0.60	−1.23	−1.74	0.23	**	0.09		0.17		0.41	**	0.35	**	0.38	**
Impersonal/Less Aversive	0.87	0.26	−0.31	0.20	**	0.04		0.23		0.36	**	0.27	**	0.43	**
Personal/More Aversive	−2.06	−2.73	−3.18	0.22	**	0.12		0.09		0.36	**	0.33	**	0.23	
Cognitive Reflection Task	2.06	1.73	1.57	0.31	**	0.18	**	0.15		0.46	**	0.38	**	0.39	**

*Note:*

* *p≤.01,*

** *p≤.001 (two tailed).*

**Table 4:** Means and Cohen's d-scores for scales in Study 3.

	Means			Cohen's d-score for Libertarians Compared to Liberals						Cohen's d-score for Libertarians Compared to Conservatives					
Scale	Libertarians	Liberals	Conservatives	Overall		Men		Women		Overall		Men		Women	
Individualism-Collectivism Scale															
Collectivism - Horizontal	3.70	4.28	4.16	−0.70	**	−0.64	**	−0.56	**	−0.55	**	−0.50	**	−0.54	**
Collectivism - Vertical	2.96	3.13	3.66	−0.22	**	−0.25	*	−0.08		−0.89	**	−0.83	**	−0.96	**
Individualism - Horizontal	5.11	4.76	4.75	0.61	**	0.67	**	0.40	**	0.59	**	0.60	**	0.50	**
Individualism - Vertical	3.69	3.08	3.66	0.65	**	0.63	**	0.53	**	0.03		−0.01		0.03	
**Identification with All Humanity Scale**															
Identification with Community	2.77	3.07	3.24	−0.36	**	−0.27	**	−0.33	**	−0.55	**	−0.55	**	−0.47	**
Identification with Country	2.94	3.01	3.60	−0.09	*	−0.02		−0.09		−0.85	**	−0.88	**	−0.74	**
Identification with World	2.69	3.41	2.64	−0.84	**	−0.78	**	−0.71	**	0.06		0.14	**	0.01	
**Different Types of Love Scale**															
Love for Family	4.64	4.79	5.02	−0.13		0.02		−0.23		−0.33	**	−0.23		−0.51	**
Love for Friends	4.97	5.24	5.10	−0.27	**	−0.14		−0.28		−0.13		−0.10		−0.14	
Love for Generic Others	4.47	5.24	4.75	−0.78	**	−0.69	**	−0.61	**	−0.28	**	−0.25	*	−0.18	
Love for Romantic Partner	5.22	5.53	5.53	−0.29	**	−0.27	**	−0.19		−0.30	**	−0.33	**	−0.18	

*Note:*

* *p<.01,*

** *p<.001 (two tailed).*

## Results and Discussion

### Study 1: Describing Libertarian Morality

*If any civilization is to survive, it is the morality of altruism that men have to reject.*

*- Ayn Rand*

Our first prediction was that, compared to liberals and conservatives, the morality of libertarians would be characterized by strong endorsement of individual liberty at the expense of other moral considerations. We addressed this question by examining several measures designed to give a broad overview of a person's values and morals, in particular the Moral Foundations Questionnaire [41], and the Schwartz Value Scale [42], as well as a new measure of endorsement of liberty as a moral principle, introduced here (see Appendix S1). For convergent validity, we also examined several other scales commonly used to measure moral orientations.

### Moral Foundations Questionnaire

The Moral Foundations Questionnaire (MFQ) measures the degree to which a person relies on each of five moral foundations: harm/care, fairness/reciprocity, ingroup/loyalty, authority/respect, and purity/sanctity. The scale has two parts. The first measures abstract assessments of moral relevance (e.g., “When you decide whether something is right or wrong, to what extent do you consider whether or not someone suffered emotionally?” for harm) and the second measures agreement with more specific moral statements (e.g., “I would call some acts wrong on the grounds that they are unnatural,” for purity). The MFQ has been shown to be reliable and valid, and to predict a variety of moral and political attitudes, independent of political ideology [41]. The MFQ was completed by 97,036 participants (54,068 men; 64,109 liberals, 13,537 conservatives, and 8,539 libertarians). The number of participants given in each section includes only those participants who self-identified as liberal, conservative, or libertarian.

#### Results

**Figure 1:**
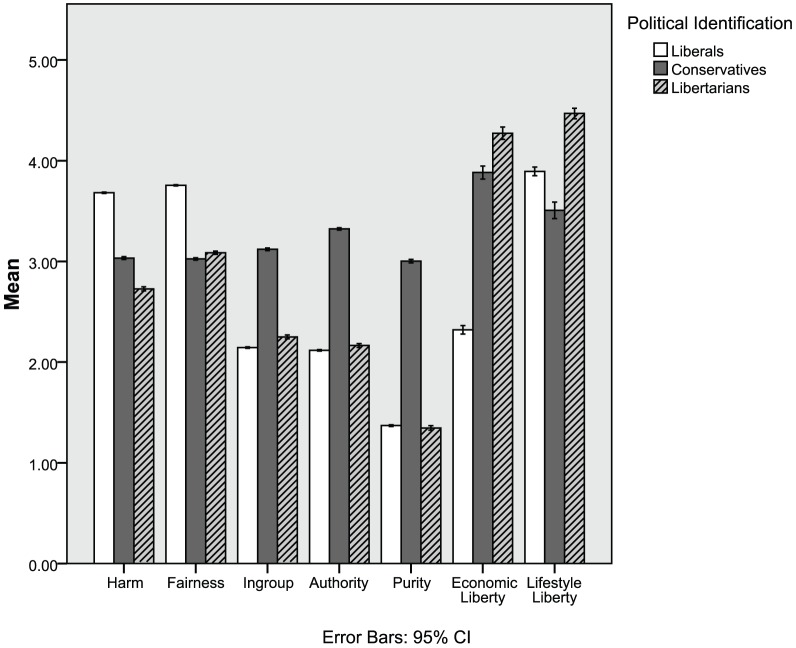
Libertarians have weaker intuitions about most moral concerns, but stronger intuitions about liberty.

The first five rows of Table 2 show *d* scores indicating how libertarians differed from liberals and conservatives on the MFQ (also see Figure 1). Libertarians were similar to conservatives on the fairness foundation, as both groups scored substantially lower than liberals. However, like liberals, libertarians scored substantially lower on the ingroup, authority, and purity foundations compared to conservatives. Finally, libertarians scored slightly lower than conservatives and substantially lower than liberals on the harm foundation. Convergent results were found using the Moral Foundations Sacredness Scale, which measures endorsement of foundations using a willingness to make tradeoffs.

#### Interpretation

Our results suggest why libertarians do not feel fully at home in either of the major American political parties. Consistent with our prediction, libertarians were relatively low on all five foundations. Libertarians share with liberals, a distaste for the morality of ingroup, authority, and purity, characteristic of social conservatives, particularly those on the religious right [43]. Like liberals, libertarians can be said to have a two-foundation morality, prioritizing harm and fairness above the other three foundations. But libertarians share with conservatives their moderate scores on these two foundations. They are therefore likely to be less responsive than liberals to moral appeals from groups who claim to be victimized, oppressed, or treated unfairly. Libertarianism is clearly not just a point on the liberal-conservative continuum; libertarians have a unique pattern of moral concerns, with relatively low reliance on all five foundations.

### Schwartz Values Scale

The SVS [42] consists of 58 statements of values. Participants rate the degree to which each value serves “as a guiding principle in his or her life,” using a 9-point scale running from “opposed to my values” to “of supreme importance.” The scale has been used widely in cross-cultural research (Schwartz et al., 2001). It produces composite scores for 10 values, which are shown in Table 2. The SVS was completed by 10,071 participants (5,426 men; 6,518 liberals, 1,278 conservatives, and 1,213 libertarians).

#### Results

Table 2 shows that libertarians are similar to liberals on most values, scoring moderately higher than conservatives on hedonism and stimulation, and substantially lower than conservatives on conformity, security, and tradition. Libertarians also scored similarly to liberals and slightly lower than conservatives on power. Libertarians departed from liberals and joined conservatives on only one value: universalism, where libertarians were substantially lower than liberals. Libertarians were unique on two values: benevolence, where they scored moderately below the other two groups, and self-direction, where they scored the highest (slightly higher than liberals and moderately higher than conservatives).

#### Interpretation

Once again, we see that libertarians look somewhat like liberals, but assign lower importance to values related to the welfare or suffering of others–the benevolence value (which Schwartz defines as: “Preservation and enhancement of the welfare of people with whom one is in frequent personal contact”) and universalism (defined as “Understanding, appreciation, tolerance, and protection for the welfare of all people and for nature”). It is also noteworthy that the highest mean for any Schwartz Value dimension was libertarians' endorsement of self-direction (defined as “Independent thought and action – choosing, creating, exploring”). Self-Direction was the most strongly endorsed value for all three groups, but for libertarians the difference was quite large compared to the next most endorsed value, achievement (*d* = 1.04). If libertarians have indeed elevated self-direction as their foremost guiding principle, then they may see the needs and claims of others, whether based on liberal or conservative principles, as a threat to their primary value.

### Ethics Position Questionnaire

The Ethics Position Questionnaire [44] is composed of two 10-item subscales measuring moral idealism and moral relativism. Idealism reflects the extent to which a concern for the welfare of others is at the heart of an individual's moral code (e.g. “People should make certain that their actions never intentionally harm another even to a small degree.”). Relativism concerns whether or not an individual believes that moral principles are universal (e.g. “What is ethical varies from one situation and society to another.”). The scale is commonly used in the business ethics literature and has been shown to predict immoral behavior in ethical situations [45]. The Ethics Position Questionnaire was completed by 8,078 participants (4,785 men; 4,991 liberals, 1,240 conservatives, and 1,001 libertarians).

#### Results

Table 2 shows that libertarians score moderately lower than liberals and slightly lower than conservatives on moral idealism. Libertarians score moderately higher than conservatives (*d* = .58), and similar but lower than liberals (*d* = −.25), on moral relativism.

#### Interpretation

According to Forsyth's [44] classification system, individuals who score high in relativism and low on idealism — the pattern found for libertarians — are labeled “subjectivists” who “reject moral rules” and “base moral judgments on personal feelings about the action and the setting.” Subjectivists have been found to be more lenient in judging individuals who violate moral norms [46]. This result is consistent with our findings on the MFQ and Schwartz Values Scale measures, in that libertarians appear to live in a world where traditional moral concerns (e.g., altruism, respect for authority) are not assigned much importance.

### Good Self Scale

The Good-Self Assessment [47] is a measure of moral self-relevance, or the degree to which one sees moral, rather than non-moral, traits as part of his/her self-concept. This is a slightly modified version of the original; for the moral traits we replaced sincere and helpful with kind and loyal, and for the non-moral traits we replaced athletic and industrious with intellectual and hardworking. In this measure participants are given a list of 8 moral and 8 non-moral positive traits (each described with two synonymous terms, e.g. “honest or truthful”) and are asked to rate their importance to their self-concept from 1 = not important to 4 = extremely important. This scale was completed by 606 participants (294 men; 367 liberals, 85 conservatives, and 77 libertarians).

#### Results

Table 2 shows that libertarians scored moderately lower than liberals and substantially lower than conservatives on the self-relevance of moral traits. They did not differ from liberals and conservatives on the importance they ascribed to non-moral traits. We also examined the non-moral term, “independent”, separately, and found that liberals (d = −.38, p<.01) and conservatives (d = −.37, p<.05) scored significantly lower than libertarians.

#### Interpretation

The results suggest that libertarians are less likely to see moral traits as important to their core self, compared to liberals and conservatives. At the same time they are just as likely as these two groups to base their self-concept around positive non-moral characteristics, such as being funny or outgoing. Notably, libertarians were the only group to report valuing pragmatic, non-moral traits more than moral traits. Libertarians may hesitate to view traits that engender obligations to others (e.g. loyal, generous, sympathetic) as important parts of who they are because such traits imply being altruistic [48].

### Lifestyle and Economic/Government Liberty

In the original conception of Moral Foundations Theory, concerns about liberty (or autonomy or freedom) were not measured. But as we began to collect data on libertarians and to hear objections from libertarians that their core value was not well represented, we created questions related to liberty in the style of the Moral Foundations Questionnaire. We generated 11 items about several forms of liberty (see Appendix S1) and collected responses from 3,732 participants (2,105 men; 2,181 liberals, 573 conservatives, and 525 libertarians). Principal component analysis using varimax rotation indicated two clear factors (Eigenvalues of 3.40 and 1.48; next highest was .74). Six items loaded greater than .60 on the first factor, which represented concerns about economic/government liberty (e.g., “People who are successful in business have a right to enjoy their wealth as they see fit”). Three items loaded greater than .60 on the second factor, which can be interpreted as a “lifestyle liberty” factor (e.g., “Everyone should be free to do as they choose, as long as they don't infringe upon the equal freedom of others.”). We created two subscales from these items (Cronbach's alpha for economic/government liberty was .81; for lifestyle liberty, .60; the correlation between factors was .27).

#### Results

Table 2 shows that libertarians scored highest on both kinds of liberty (also see Figure 1). On economic/government liberty, liberals were the outliers, scoring below the midpoint of the scale, two full standard deviations below libertarians (*d* = 2.56). On lifestyle liberty, libertarians scored substantially higher than both liberals (*d* = .81), and conservatives (*d* = 1.19).

#### Interpretation

Libertarians are not unconcerned about all aspects of morality, as suggested by their scores on the MFQ and several other widely used morality scales. Rather, consistent with their self-descriptions, they care about liberty. Like conservatives, they endorse a world in which people are left alone to enjoy the fruits of their own labor, free from government interference. They also exceed both liberals and conservatives (but are closer to liberals) in endorsing personal or lifestyle liberty.

### Do libertarians have a unique moral profile?

We conducted two analyses to answer this question, in addition to the above comparisons. First, we conducted a cluster analysis of participants using Moral Foundations Questionnaire sub-scale scores, to see if we could statistically extract libertarians based on their pattern of responses concerning their values, rather than on their self-identification. Second, we conducted a principal components analysis of the measures included in Study 1 in order to see if the values that libertarians espouse did indeed form a coherent factor.

#### Cluster Analysis

A hierarchical cluster analysis was conducted on all participants who completed both the basic Moral Foundations Questionnaire sub-scale scores, as well as the Liberty Foundation scores (N = 3,094), using Ward's Method to calculate the distance between participants. Visual analysis of the resulting dendogram indicated that a major third cluster split occurs at a significant distance from further divisions, and we therefore classified all participants based on this three-cluster solution. Mean scores and the composition of each group are given in Table 1. Group 1, in which 74% of participants self-identified as liberal, shows a high concern for harm, fairness, and lifestyle liberty. Group 2, in which a plurality (44%) self-identified as conservative, shows a more even distribution of concerns across all moral foundations. Group 3, in which 60% self-identified as libertarian, shows by far the highest concern for lifestyle and economic/government liberty, and the lowest level of concern on the five moral foundations.

#### Principal Components Analysis

**Figure 2:**
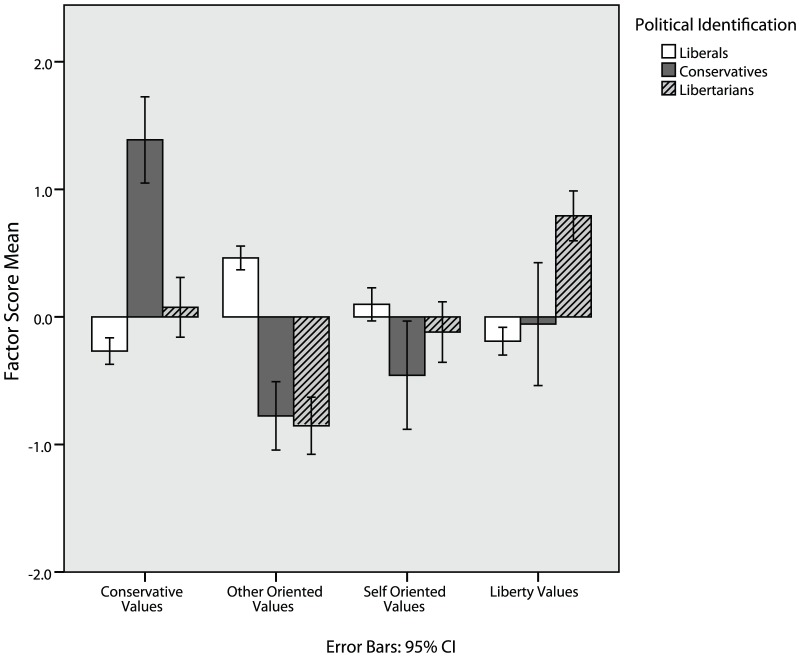
Libertarians are more concerned with liberty values and less concerned with other-oriented and conservative values.

Principal components analysis using all measures from Study 1, except for the Good-Self Scale, was conducted on 374 participants (214 liberals, 31 moderates, 31 conservatives, 72 libertarians, and 26 other/apolitical) who completed these measures. Scree plot analysis [49] indicated a 4 factor solution was appropriate, with only four factors having an eigenvalue greater than one. Four factors were extracted using varimax rotation, which we interpreted as conservative values (e.g. MFQ-purity), other-oriented values (e.g. MFQ-harm), self-oriented values (e.g. Schwartz Values-power), and liberty values (including measures of lifestyle liberty, economic/government liberty, and Schwartz Values-self direction). Table 5 lists all factor loadings greater than .10. Standardized factor scores were computed for each participant and analyzed across political groups (Figure 2), indicating that libertarians are indeed characterized by liberty values, conservatives by conservative values, and liberals by other-oriented values. Convergent results were found with the Good Self Subscales included, with Moral Traits loading on other-oriented values and pragmatic traits loading on self-oriented values, but the sample size (N = 79) is below what is customary in factor analyses.

**Table 5:** Principle Components Analysis Factor Loadings of Variables in Study 1.

Variable Name	Conservative Values	Other- Oriented Values	Self-Oriented Values	Liberty Values
MFQ Purity	**.790**		−.176	
Schwartz Values Conformity	**.782**	.184	.184	
Schwartz Values Tradition	**.772**	.214		
MFQ Authority	**.769**	−.239		−.257
MFQ Ingroup	**.652**	−.176	.119	−.187
Schwartz Values Security	**.618**	.182	.446	.196
EPQ - Relativism	−.456		.273	
MFQ Harm		**.857**		
Schwartz Values Universality		**.851**	.204	
MFQ Fairness	−.110	**.771**		
EPQ - Idealism	.178	**.691**	−.104	.121
Schwartz Values Benevolence	.413	**.668**	.242	.228
MFQ Economic Liberty	.338	**−.620**	−.106	**.568**
Schwartz Values Power	.166	−.220	**.754**	−.251
Schwartz Values Achievement	.167	.153	**.735**	.102
Schwartz Values Stimulation	−.185	.155	**.562**	.276
Schwartz Values Hedonism	−.317		**.560**	.324
MFQ Lifestyle Liberty	−.308			**.755**
Schwartz Values Self Direction		.197	.432	**.696**

Note: Factor loadings <|.1| omitted. Factor loadings >|.5| bolded.

#### Interpretation

The above analyses suggest that libertarians indeed hold an empirically distinct set of values, compared to liberals and conservatives. Given that liberty values form an empirically distinct value cluster that has pragmatic utility in differentiating groups and is distinct from other self-oriented concerns such as power and achievement, it is likely that concerns about liberty represent a moral intuition previously unmeasured in Moral Foundations Theory. A cluster analysis of participants yielded a 3-group solution where members of this third group endorsed libertarian values more and liberal/conservative values less, and were also more likely to be libertarian (see Table 1). Principal components analysis yields a distinct “liberty values” factor that meaningfully differentiates individuals. Patterns of endorsement across all four components indicate that libertarians have a moral profile that is clearly distinct from both liberals and conservatives (see Figure 2). Libertarians generally score over a half a standard deviation lower than liberals on variables which compose the “other-oriented values” factor and over a half a standard deviation lower than conservatives on variables which load on the “conservative values” factor (see Table 1). Libertarian scores are similar to those of liberals on “self-oriented values.” Finally, libertarians score higher than both liberals and conservatives on “liberty values.”

### Study 1 Summary: What is Libertarian Morality?

Our results suggest that libertarians are a distinct group that places lower value on morality as typically measured by moral psychologists. This pattern was replicated across a variety of largely separate samples with moral concerns measured using several different approaches. Our measures were not overtly political in content, and there were few questions about the role of government. Rather, we used measures of general values and moral beliefs, and found that libertarians were consistently less concerned than other groups about the individual-level, other-oriented concerns that most theorists place at the heart of morality: harm, benevolence, and altruism. The contrast here was starkest with liberals, but we also found that libertarians were much less concerned than conservatives with group-level moral issues (e.g. conformity, loyalty, and tradition) that are typically associated with conservative morality [3]. Libertarians viewed commonly measured moral traits, but not pragmatic traits, as less essential to their self-concept.

This is not to say, however, that libertarians are devoid of moral concerns. Contemporary moral psychology has paid little attention to the valuation of negative liberty as a specifically moral concern. Independence may be seen as a pragmatic value [47]. Respecting the autonomy of others may be seen as a way to promote the welfare of individuals [43], consistent with liberal ideas about positive liberty, rather than as an independent moral construct. It is predictable, then, that on such measures libertarians appear amoral (i.e. lacking in the activation of common moral systems). However, our results show that libertarians score substantially higher than liberals and conservatives on measures of both economic and lifestyle liberty, the Schwartz value of Self-Direction, and the centrality of independence to one's core self (measured using the Modified Good Self scale). Libertarians may fear that the moral concerns typically endorsed by liberals or conservatives (as measured by the MFQ) are claims that can be used to trample upon individual rights — libertarians' sacred value (e.g. [48]). If liberty is included as a moral value, libertarians are not amoral. Rather, standard morality scales, including the Moral Foundations Questionnaire, do a poor job of measuring libertarian values.

Therefore, our first prediction was strongly supported: libertarians value liberty more strongly and consistently than liberals or conservatives, at the expense of other moral concerns. We now turn to the question of libertarian dispositions. In particular, might libertarians simply feel the emotional pull of most moral concerns more weakly than other people do? Might libertarians generally be dispositionally more rational and less emotional? Study 2 tests whether these dispositional traits (level 1) may lead libertarians to certain values (level 2) and then to the endorsement of certain ideological narratives (level 3), which tie these values together in the form of an ideology [9].

### Study 2: How Do Libertarians Think and Feel?

*“Every aspect of Western culture needs a new code of ethics - a rational ethics - as a precondition of rebirth.”*

*- Ayn Rand* [50]

In Study 2, we sought to examine cognitive and emotional differences among libertarians, liberals, and conservatives. Psychologists have long theorized that values evolve from the interaction of heritable dispositions, childhood learning, and social-contextual factors [34], [51]. We expected the libertarian dispositional profile to converge with the results of Study 1, in which libertarians showed a relative lack of concern for the most common moral considerations. Given the well-documented influence of emotions on moral judgment and behavior [24], [52]–[54], if it turns out that libertarians feel fewer or weaker moral emotions, then it is understandable that their morality would be substantially different from that of liberals and conservatives.

In place of a system of morality deriving from emotion, libertarians have explicitly sought a “rational ethics” [28]. Among the main traits that have been found to distinguish liberals from conservatives are those related to cognitive style. Liberals score higher on traits related to tolerance for ambiguity, need for cognition, and openness to experience [4]. Based on the explicitly intellectual focus of libertarian writing, and on their general lack of concern for tradition and traditional morality, we expected that libertarians would generally resemble liberals on such measures.

These considerations led us to our second prediction: Libertarians will reveal a cognitive style that depends less on emotion– and more on reason– than will either liberals or conservatives. We expected this cognitive style to relate to the distinct moral profile described in Study 1, leading to libertarian self-identification.

### Big Five Personality Inventory

The Big Five Personality Inventory [55] is a 44-item measure of five personality traits often said to be the most fundamental traits in personality psychology: openness to experience, conscientiousness, extraversion, agreeableness, and neuroticism. The measure was completed by 29,043 participants (14,091 men; 19,106 liberals, 3,991 conservatives, and 2,615 libertarians).

#### Results

Table 3 shows that libertarians scored lower than the other two groups on agreeableness, conscientiousness, and extraversion. They scored low (similar to conservatives) on neuroticism, and they scored quite high (similar to liberals) on openness to experience.

#### Interpretation

The libertarian pattern on the Big 5 complements our findings on their explicit values in Study 1. Libertarians report lower levels of the traits that indicate an orientation toward engaging with and pleasing others (i.e., extraversion and agreeableness). Low scores on agreeableness in particular have been said to indicate a lack of compassion and a critical, skeptical nature [51]. In addition, as in Study 1, we see that libertarians share traits with liberals (high openness to experience) as well as conservatives (low neuroticism).

### Interpersonal Reactivity Index

The Interpersonal Reactivity Index (IRI [56]) is a 28-item measure of empathy, with 7 items covering each of four distinct aspects of empathic responding to others: 1) empathic concern for others, 2) fantasy, 3) personal distress, and 4) perspective-taking. Participants were asked whether certain statements did or did not characterize them very well (e.g. “I often have tender, concerned feelings for people less fortunate than me,” for empathic concern). The IRI was completed by 6,450 participants (3,073 men, 4,103 liberals, 906 conservatives, and 697 libertarians).

#### Results

**Figure 3:**
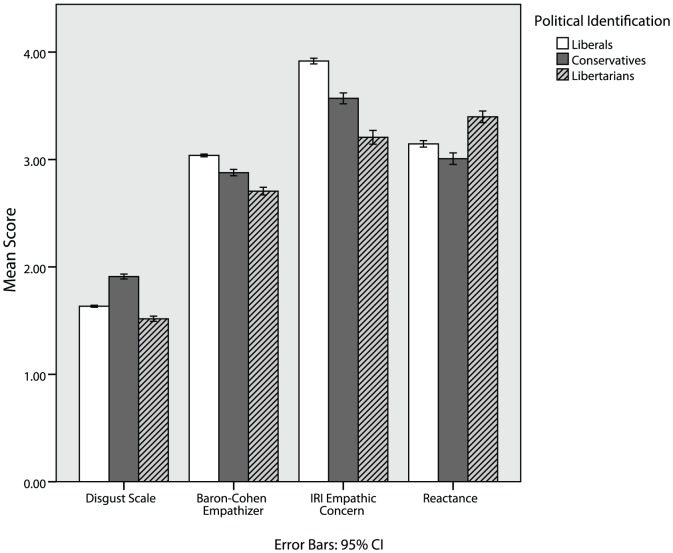
Libertarians report lower emotional responsiveness, but higher levels of psychological reactance.

Table 3 shows that libertarians scored moderately lower than conservatives and substantially lower than liberals on empathic concern for others (also see Figure 3). Libertarians score slightly lower than liberals and similar to conservatives on personal distress, perspective taking, and fantasy.

#### Interpretation

According to Davis [56], low levels of empathic concern indicate lower levels of sympathy and concern for unfortunate others, which may underlie libertarians' lower scores on the harm foundation of the MFQ, and their general rejection of altruism as a moral duty.

### Disgust Scale

The Disgust Scale Revised [57], [58] measures individual differences in the propensity to feel disgust toward three classes of elicitors: 1) core disgust (animals and body products that pose a microbial threat, such as rats, vomit, and dirty toilets); 2) animal-reminder disgust (corpses, gore, and other reminders that human bodies are mortal, like animal bodies); and 3) contamination (concerns about coming into physical contact with other people). The measure was completed by 32,738 participants (16,477 men; 23,516 liberals, 3,617 conservatives, and 2,368 libertarians).

#### Results

Table 3 shows that libertarians scored moderately lower than conservatives and slightly lower than liberals (also see Figure 3). However, the comparison to liberals appears to be driven by the fact that libertarians tend to be male and men tend to have lower levels of disgust sensitivity [57]. Within each gender, libertarians and liberals score similarly on the disgust scale. In contrast, libertarians score moderately lower than conservatives on measures of disgust within both genders (see Table 3) and across all three classes of disgust.

#### Interpretation

Previous research has shown that liberals are less disgust-sensitive than conservatives [14]. The low level of disgust sensitivity found in libertarians is consistent with previous research about the relationship between disgust and conservative attitudes on social issues, particularly those related to sexuality (e.g. MFQ-Purity in Study 1). Libertarians may not experience the flash of revulsion that drives moral condemnation in many cases of unorthodox behavior [59].

### Hong Reactance Scale

The Hong Reactance scale [60] is an 11-item measure of psychological reactance [61]. The scale measures the extent to which people are emotionally resistant to restrictions on their behavioral freedom and to the advice and influence of others. The measure was completed by 3,685 participants (1,777 men, 2,301 liberals, 510 conservatives, and 445 libertarians).

#### Results

Table 3 shows that libertarians score slightly higher than liberals and moderately higher than conservatives on psychological reactance (also see Figure 3).

#### Interpretation

The high levels of reactance expressed by libertarians fit well with the value they place on liberty as a moral foundation. It is of course possible that libertarians' responses to the scale are primarily expressions of their current political beliefs, but it is also possible that people who have the strongest visceral reactions to interference from others are also the people most drawn to the ideals and identity of libertarianism. Reactance may in fact function as a moral emotion that draws individuals toward the ideal of negative liberty. Reactance scores were negatively correlated with measures of empathy (Big Five Agreeableness: r = −.38, Baron-Cohen Empathizer: r = −.32, IRI Empathic Concern: r = −.15; p<.001 in all cases) that are most associated with conceptions of positive liberty [18], which perhaps suggests why, in the US, libertarianism is more commonly associated with conservative, as opposed to liberal policies.

### Empathizer-Systemizer Scale

The Empathizer-Systemizer scale (adapted from Baron-Cohen [62]) measures the tendency to empathize, defined as “the drive to identify another person's emotions and thoughts, and to respond to these with an appropriate emotion” and to systemize, or “the drive to analyze the variables in a system, and to derive the underlying rules that govern the behavior of the system.” In short, empathizing is about understanding the social world whereas systemizing is about understanding the world of inanimate objects and nature. We selected 20 items from the full 40-item empathizer scale, and 20 items from the full 75-item systemizer scale to create a single survey that could be completed in less than 10 minutes. Cronbach's alphas for these measures were .80 (systemizer) and .84 (empathizer). The measure was completed by 8,870 participants (4,532 men, 6,525 liberals, 877 conservatives, and 637 libertarians).

#### Results

**Figure 4:**
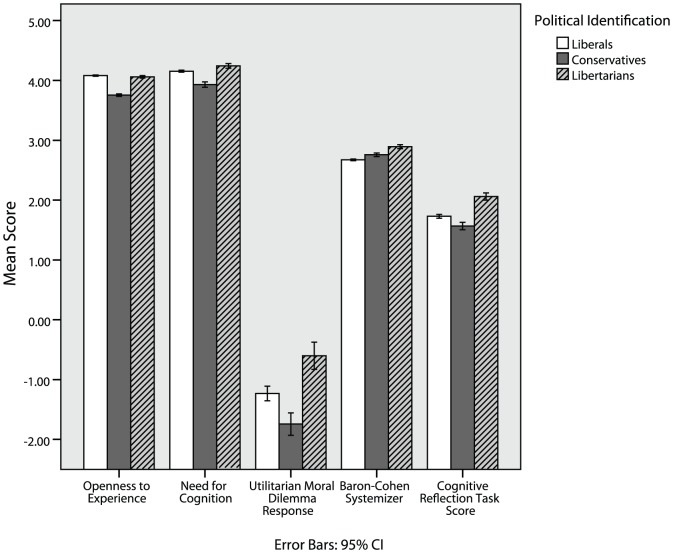
Libertarians exhibit a reason-based cognitive style according to a variety of measures.

Table 3 shows that libertarians score the lowest of any group on empathizing, and the highest on systemizing (also see Figures 3 and 4). In fact, libertarians are the only group that scored higher on systemizing than on empathizing. Given that these traits are known to differ between men and women, it is important to examine these effects in each sex separately. Table 3 shows that the same effects hold when looking only at men, and when looking only at women.

#### Interpretation

Research by Baron-Cohen [62] has shown that relatively high systemizing and low empathizing scores are characteristic of the male brain, with very extreme scores indicating autism. We might say that liberals have the most “feminine” cognitive style, and libertarians have the most “masculine.” These effects hold even when men and women are examined separately, as can be seen in Table 3. Indeed, the “feminizing” of the Democratic party in the 1970s [63] may help explain why libertarians moved increasingly into the Republican party in the 1980s.

### Need for Cognition

The Need for Cognition scale [64] is a measure of the extent to which people engage in and enjoy effortful cognitive activities. People with high need for cognition are more likely to form their attitudes by paying close attention to relevant arguments, whereas people with low need for cognition are more likely to rely on peripheral cues, such as how attractive or credible a speaker is. The measure was completed by 8,035 participants (4,242 men; 5,888 liberals, 760 conservatives, and 657 libertarians).

#### Results

Table 3 shows that libertarians scored slightly higher than liberals and moderately higher than conservatives on Need for Cognition (also see Figure 4).

#### Interpretation

This pattern is consistent with the libertarian valuation of logic and reasoning over emotion. Libertarians may enjoy thinking about complex and abstract systems more than other groups, particularly more than conservatives.

### Moral Dilemmas

Six moral dilemmas adapted from Greene et. al. [24] were given to each participant. Each dilemma required a choice about whether to take an action to save multiple individuals at the cost of a single individual's life. Each dilemma was modified so that there was one more aversive version (e.g. “push this stranger off the bridge and onto the tracks below, where his large body will stop the trolley” – called “personal” in Greene et. al., [24]) and one less aversive version (e.g. “hit the switch, which will cause the trolley to proceed to the right”). Participants were randomly assigned to receive one version of each dilemma; each participant received three aversive and three less aversive dilemmas. Below the dilemma text was the question “Is it morally appropriate for you to [do action] in order to [prevent some other danger]?” with a dichotomous No/Yes response option. These questions were followed by the question “How certain are you about your answer?” with a 7-point response scale from “extremely uncertain” to “extremely certain.” Participants' responses to the 12 dichotomous choice questions were weighted by certainty and then averaged, with higher scores indicating greater willingness to make utilitarian sacrifices. The measure was completed by 4,629 participants (2,615 men; 2,690 liberals, 765 conservatives, and 616 libertarians).

#### Results

Table 3 shows that libertarians were moderately more utilitarian than conservatives, and slightly more utilitarian than liberals (also see Figure 4). Their judgments were more utilitarian in both the more aversive and less aversive scenarios.

#### Interpretation

The results from these moral dilemmas, which are devoid of political content, indicate that libertarians are indeed more capable of “rational ethics” where costs and benefits are weighed according to utilitarian principles. Given the body of evidence suggesting that utilitarian judgments in these dilemmas are more likely to be reached via “cold” calculation, and that deontological (rights-based) judgments are more likely to be reached via “hot” affective processes (e.g., [24], [65]), our results suggests that libertarians are particularly unemotional in their moral deliberations.

### Cognitive Reflection Task

The Cognitive Reflection Task [66] is a set of 3 logic questions that have correct and intuitive answers. Correct answers on these questions is said not just to measure intelligence, but also to measure a person's ability to suppress an intuitive response in service of the cognitive reasoning required to solve these problems. The measure was completed by 9,721 participants (4,971 men; 7,384 liberals, 1,267 conservatives, and 1,070 libertarians).

#### Results

Table 3 shows that libertarians find the correct answers to these questions at a slightly higher rate than liberals and moderately higher rate compared to conservatives (also see Figure 4).

#### Interpretation

The cognitive reflection task provides a behavioral validation of the hypothesis that libertarians have a more reasoned cognitive style. In our dataset, this measure inter-correlates with both Need for Cognition (r = .30, p<.001) and Baron-Cohen Systemizer (r = .31, p<.001) scores, with libertarians scoring higher than both liberals and conservatives on all three measures. Taken together, a convergent picture of the rational cognitive style of libertarians emerges (Figure 4).

### Do libertarian dispositions lead to libertarian values?

Consistent with McAdams' personality model [34], previous research has found that dispositions predispose individuals to moralize specific concerns, which in turn constrain ideological choice [9]. We examined a model in which dispositional effects on ideological identification are mediated by value orientations, as measured by the Moral Foundations Questionnaire with questions concerning liberty added. This model has been previously found to be a superior fit to similar data, in comparison to alternative models [9]. Following the model previously used by Lewis & Bates [9], we examined Individualizing (indicated by MFQ-Harm & Fairness) and Binding (indicated by MFQ-Ingroup, Authority, & Purity) values as related to disgust sensitivity and empathy, two key dispositional constructs identified in previous research as being related to these respective values [67], [68]. We also included the lone positive dispositional explanation for the libertarian valuation of negative liberty (psychological reactance) to the Study 1 values most endorsed by libertarians (economic/government and lifestyle liberty). The dependent measure was a dichotomous variable, self-identification as libertarian.

**Figure 5:**
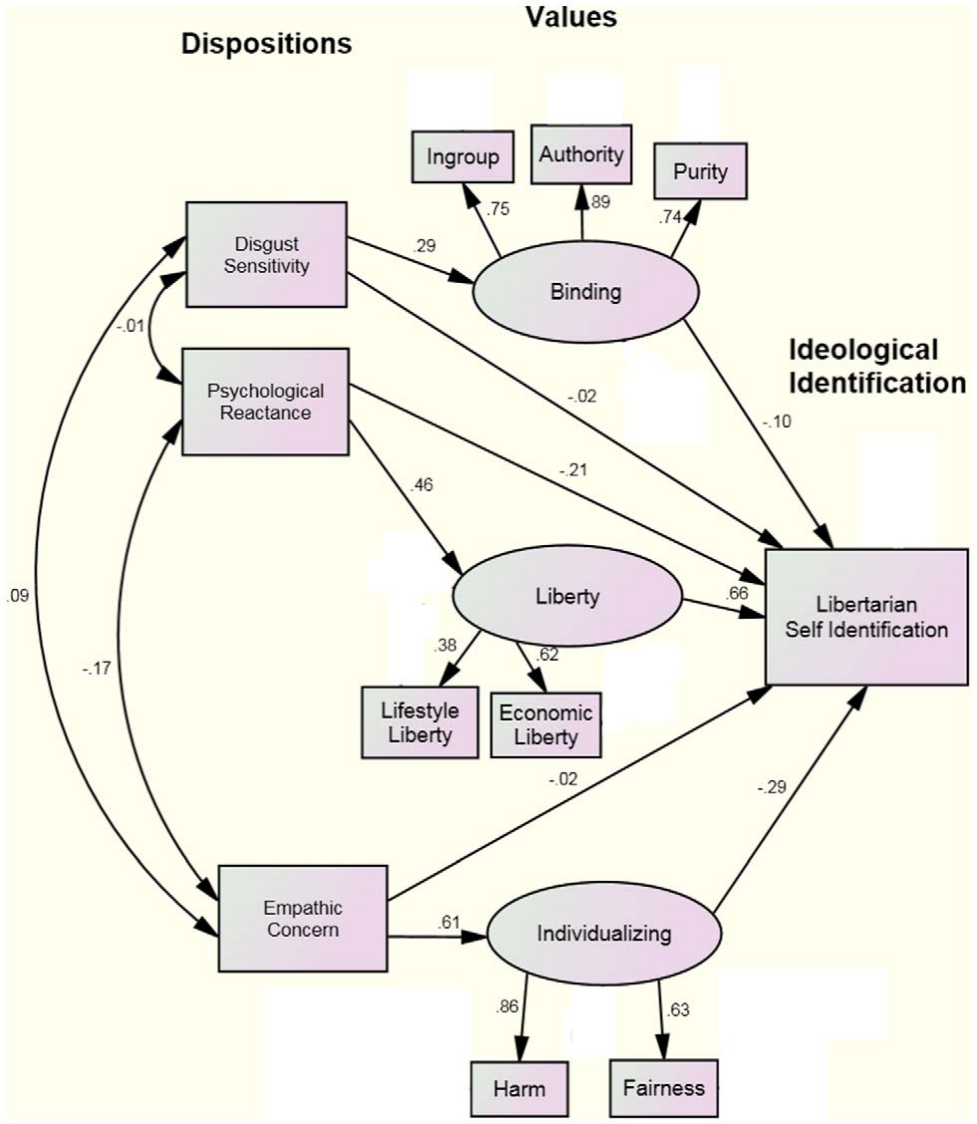
Structural Equation Model showing relationship between libertarian dispositions and values.

Using AMOS 19, three structural equation models were created and compared: 1. a partial mediation model whereby dispositional variables affect libertarian self-identification directly as well as mediated by values (see Figure 5), 2. a full mediation model whereby dispositional variables could only affect libertarian self-identification by influencing values variables (the same model as Figure 5, except that the paths connecting Disgust Sensitivity, Psychological Reactance, and Empathic Concern directly to libertarian self-identification were removed), and 3. an independence model whereby dispositional and values variables separately influence libertarian self-identification (the same model as Figure 5, except that paths connecting Disgust Sensitivity to Binding values, Psychological Reactance to Liberty values, and Empathic Concern to Individualizing values, were all removed). Both the partial mediation (RMSEA = .044, Chi-Squared = 11028.75 (df = 36, p<.001), CFI = .94) and full mediation models (RMSEA = .042, Chi-Squared = 11062.75 (df = 39, p<.001), CFI = .94) were good fits to the data. Given our large sample size, the change in goodness of fit statistic is likely to be more diagnostic of model fit compared to statistics such as Chi-Squared [69]. Using this criteria, the independence model was a comparatively worse fit to the data (RMSEA = .049, Chi-Squared = 15000.14 (df = 39, p<.001), CFI = .92). Parsimony would suggest selecting the full mediation model, and examining the regression weights estimated in the partial mediation model (Figure 5) to compare the direct path to libertarian self-identification versus the mediated paths for Disgust Sensitivity (.02 vs. .29/−.10), Psychological Reactance (−.21 vs. .46/.66), and Empathic Concern (−.02 vs. .61/−.29), suggests that direct effects are relatively small.

We also tested these mediation models using a procedure developed by Baron & Kenny [70], adapted for a dichotomous outcome [71], and the results converged with the results of these SEM analyses in that a significant (Sobel Test, p<.01 in each case) percentage of the relationship between dispositions and libertarian self-identification was mediated by values in each path. However, more variance in libertarian self-identification was mediated via the Empathic Concern->Individualizing Values path (56%) and Psychological Reactance-Liberty Values path (76%) compared to the Disgust Sensitivity-Binding Values path (11%). Overall, consistent with the results that Lewis & Bates [9] found using liberals and conservatives, the effect of dispositional variables on libertarian self-identification is largely mediated by related values.

### Study 2 Summary: How Do Libertarians Think and Feel?

As predicted, libertarians showed lower levels of emotional responsiveness on standard measures of the moral emotions of disgust and empathy (Figure 3). Multivariate analyses indicate that, consistent with McAdams' personality model and previous research on these moral emotions, these dispositions relate to values, in ways which may predispose some individuals to choose to identify as libertarian. From an intuitionist perspective, libertarians' relative lack of emotional reactions may help explain the generally low levels of moral concern that we found in Study 1 (see also [25]). McCrae and Costa [51] argue that low levels of neuroticism, agreeableness, and extraversion are indicative of an unemotional style. Libertarians were the only group to report a more systematic, rather than empathic, way of understanding the world, a characteristic of men [62] that may explain why libertarianism appeals to men more than women. If morality is driven largely by emotional reactions, and if libertarians are less emotional on most of the measures we examined, then libertarians should be moved by fewer moral concerns, as was the case in Study 1.

Libertarians did display high scores, however, on one measure of emotional reactivity, the Hong Reactance scale (Figure 3), which was found to lead to libertarian values and ideological identification. This pattern is quite consistent with the pattern of moral evaluations expressed in Study 1 where libertarians' low valuation of traditional moral concerns contrasted sharply with the uniquely high moral value they placed on liberty. Libertarians also reported lower levels of agreeableness, measured using items such as “likes to cooperate with others,” and related to psychological reactance [72]. Psychological reactance may provide an intuitionist explanation [8] for the libertarian moralization of liberty.

The use of liberty rhetoric may have different psychological origins in different political groups. Autonomy is posited to be a universal basic human psychological need [73], and thus liberals may be attracted to liberty as a means of improving the psychological welfare of individuals. Similarly, social conservatives may be attracted to liberty as a means toward opposing redistributive taxation policies that challenge the status quo, yet still feel comfortable with the lifestyle liberty constraints that tradition and conformity require (see [22] for an explanation of this inconsistency). In contrast, libertarians may not see liberty as a means, but rather as an end, in and of itself, based on their heightened feelings of psychological reactance. The idea that libertarians are dispositionally more reactant than others when confronted with societal constraints is a potential gut-level explanation for their moralization of liberty. It is also evident in libertarians' fondness for the historical phrase “Don't Tread on Me,” which became a slogan of Ron Paul's 2008 presidential campaign and is frequently displayed on signs and flags at rallies for Tea Party supporters.

Consistent with their stated preference for rationality, libertarians seem to enjoy effortful and thoughtful cognitive tasks (Figure 4). In combination with low levels of emotional reactivity, the highly rational nature of libertarians may lead them to a logical, rather than emotional, system of morality, explaining their unique pattern of scores on the moral psychology measures used in Study 1. This logical system of morality may have led libertarians to be able to provide correct, rather than intuitive, answers on the cognitive reflection task, and to make more utilitarian judgments in the moral dilemmas presented to them in Study 2. Libertarians report being relatively open to new experiences and desiring stimulation, yet given the pattern of results from this study, it is likely true that libertarians may prefer intellectually stimulating experiences over emotionally stimulating experiences (e.g. social experiences). We examine this idea further in Study 3.

In conclusion, we found strong support for our second prediction, that libertarians will rely upon emotion less – and reason more – than will either liberals or conservatives. Further, multi-variate models suggest that these emotional differences may lead to certain value orientations which in turn predispose individuals toward libertarian self-identification. In the next section we explore how these value orientations may also have roots in specific patterns of (and attitudes about) social relationships, consistent with theories about the social function of moral reasoning [17], [29], [30], [33].

### Study 3: How Do Libertarians Relate to Others?

“*To say ‘I love you’ one must first be able to say the ‘I.’”*

*- Ayn Rand (1943)*

One of the primary purposes of moral concerns such as conformity, tradition, authority, and group-loyalty is to bind individuals together [31]. The same areas of the brain that are essential to normal, implicit, intuitive moral reasoning have also been found to be essential for navigating the human social world [74]. The libertarian endorsement of the liberty principle might be related to their lower levels of agreeableness and higher levels of psychological reactance, but it could also result, in part, from lower levels of extraversion, and a desire to be free of the constraints that relationships often entail. Libertarians may be members of an ultra-social species who prefer less social connection than their liberal and conservative peers.

Study 3 tests the idea that *libertarians will be more individualistic and less collectivist compared to both liberals and conservatives*, suggesting that their moral concern for liberty may represent the conversion of this preference into a value [10]. Moral concerns exist, at least in part, to serve one's own desire for social connection [33], and it therefore would logically follow that a group that has less desire for social connection would also have fewer moral concerns. To that end we assessed libertarians' sense of interconnectedness and their love for close others, such as friends, family, and romantic partners, as well as their attachments to abstract entities like one's community, country, and the world. We examined whether libertarian patterns of individualism and collectivism do indeed relate to their moral profile, predisposing them toward the libertarian ideology.

### Individualism-Collectivism

The Individualism-Collectivism scale [75] is a 32-item scale that measures an individual's levels of independence vs. interdependence. Individualists tend to emphasize self-reliance, independence and (sometimes) competition. There are two types of individualism: horizontal individualism reflects a belief that people are separate (independent) but equal entities (e.g. “I am a unique individual”), and vertical individualism emphasizes hierarchy and competitiveness between those separate entities (“It is important that I do my job better than others”). Collectivists, on the other hand, tend to emphasize cooperation, and (sometimes) equality. As with individualism, there are two kinds of collectivism, a more egalitarian (horizontal) dimension (e.g. “The well-being of my coworkers is important to me.”) and a more hierarchical (vertical) one (e.g. “Children should be taught to place duty before pleasure.”). The measure was completed by 2,975 participants (1,468 men; 1,987 liberals, 390 conservatives, and 291 libertarians).

#### Results

Table 4 shows that libertarians scored lowest on both forms of collectivism, and highest on horizontal individualism, while matching conservatives on their high scores (relative to liberals) on vertical individualism.

#### Interpretation

Libertarians appear more individualistic and less collectivistic than both liberals and conservatives. The relative preference for individualism occurs in both hierarchical and non-hierarchical circumstances.

### Identification with All of Humanity

The Identification with All of Humanity Scale [76] is a 27-item measure of connection to people in one's community, one's country, and the world. It asks 9 questions concerning each of these three groups (e.g. “How much would you say you have in common with the following groups?”). The measure was completed by 12,503 participants (7,334 men; 8,219 liberals, 1,667 conservatives, and 1,450 libertarians).

#### Results

Table 4 shows that libertarians are less identified with their community compared to both liberals and conservatives. They also scored low (just below liberals) on identification with country, which was the dimension that conservatives most strongly endorsed. In addition, they scored low (equal to conservatives) on identification with people all over “the world,” which was the dimension that liberals most strongly endorsed.

#### Interpretation

Consistent with the libertarian desire for personal liberty, libertarians feel relatively low levels of connection to their community, country, and people globally. This pattern suggests that libertarians are likely to join conservatives in opposing transnational humanitarian undertakings, and they are likely to join with liberals in opposing projects and legislation that are aimed at strengthening national identity.

### Different Types of Love scale

The Different Types of Love scale [77] is a 40-item measure of loving feelings toward four different groups. Participants indicate agreement with statements concerning friends (e.g., “The connection I feel to my friends is strengthened by all we have in common”), family (“My Mom and/or Dad's acts of unconditional love fill me with strong feelings of love”), generic others (“Doing kind things for others is a reward in itself”), and their romantic partner (“I feel love whenever anything reminds me of my partner”; participants are asked to skip all questions that do not apply). Cronbach's Alpha for each sub-scale of the Different Types of Love scale were .80 (friends), .85 (family), .87 (generic others), & .82 (romantic partners). The measure was completed by 2,776 participants (1,437 men; 1,894 liberals, 325 conservatives, and 310 libertarians).

#### Results

Table 4 shows that libertarians showed the lowest levels of loving feelings toward others, across all four categories (although the difference with conservatives on love for friends was not significant).

#### Interpretation

Consistent with the results on the Identification with All of Humanity scale, the libertarian independence from others is associated with weaker loving feelings toward friends, family, romantic partners, and generic others. It is noteworthy that differences between liberals and conservatives were generally small (except toward generic others). Libertarians were the outliers.

### Does libertarian individualism relate to libertarian values?

In order to answer this question, we conducted a principal component analysis to determine if these variables could be grouped into common ‘sociality’ factors and if libertarians do indeed score low on them. Further, we wanted to relate these factors to value clusters from Study 1.

#### Principal Component Analysis

**Figure 6:**
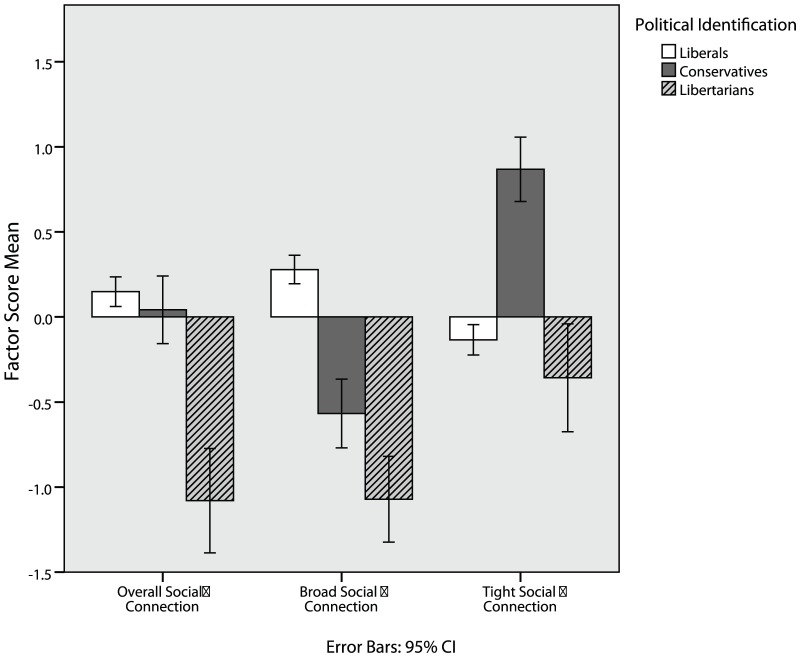
Libertarians are less connected to others, including both broad and tight social connections.

Principal component analysis using the Individualism-Collectivism Scale, Identification with All Humanity Scale, and Different Types of Love Scale was conducted on 630 participants who completed all three measures. The scree plot [49] indicated a 2 factor solution was appropriate, including a group of broad connection, more universalist oriented variables (e.g. love of friends, identification with the world) which are more typical of liberals, and a group of tight connection, close group oriented variables (e.g. love of family, identification with country) which are more typical of conservatives. Factor loadings for all variables in Study 3 are listed in Table 6. Figure 6, using standardized factor scores extracted for each participant, shows that libertarians have both lower levels of broad social connection and lower levels of tight social connection.

**Table 6:** Principle Components Analysis Factor Loadings of Variables in Study 3.

Variable Name	Broad Social Connection	Tight Social Connection
Love of Generic Others	**.851**	.231
Identification with World	**.757**	
Horizontal Collectivism	**.675**	.492
Vertical Individualism	**−.663**	.370
Identification with Community	**.580**	**.514**
Love of Friends	**.530**	.348
Horizontal Individualism	−.262	−.182
Vertical Collectivism		**.725**
Love of Family		**.606**
Identification with Country	.395	**.602**
Love of Romantic Partner	.353	.405

*Note: Factor loadings <|.1| omitted. Factor loadings >|.5| bolded.*

Of these participants, 590 also completed the Moral Foundations Questionnaire. Variables indicative of “Other Oriented Values” correlated positively with the broad social connection factor (MFQ-harm: *r* = .60, *p*<.001, MFQ-fairness: *r* = .42, *p*<.001, MFQ-authority: *r* = −.18, *p*<.001, MFQ-ingroup: *r* = −.14, *p*<.01, MFQ-purity: *r* = −.07, *p* = .07), while variables indicative of “Conservative Values” correlated positively with the tight social connection factor (MFQ-harm: *r* = .02, *p* = .61, MFQ-fairness: *r* = .01, *p* = .85, MFQ-authority: *r* = .51, *p*<.001, MFQ-ingroup: *r* = .50, *p*<.001, MFQ-purity: *r* = .44, *p*<.001).

#### Interpretation

Factor analyses indicate that the variables in Study 3 can be grouped into measures of tight social connection and measures of broad social connection. Libertarians score lower on both of these factors (Figure 6). If we relate Moral Foundation Questionnaire variables to these factors, we find that the values that typify liberals (MFQ-harm and MFQ-fairness) relate to this first factor, while the values that typify conservatives (MFQ-authority, ingroup, and purity) relate to this second factor. This is evidence for the idea that “moral thinking is for social doing” [33], as the moralities of liberals and conservatives empirically relate to the types of relationships and identifications that they seek. Notably, libertarians report lower valuation of both typically liberal and conservative concerns (Figures 1 and 2) and correspondingly lower connectedness to the groups that typically are connected to either liberals or conservatives (Figure 6).

### Study 3 Summary: How Do Libertarians Relate to Others?

As predicted, libertarians in our sample appeared to be strongly individualistic. Compared to liberals and conservatives, they report feeling a weaker sense of connection to their family members, romantic partners, friends, communities, and nations, as well as to humanity at large. While liberals exhibit a horizontal collectivistic orientation and conservatives a vertical collectivistic orientation, libertarians exhibit neither type of collectivism, instead displaying a distinctly individualistic orientation. This relative preference for individualism may have been moralized [10] into the value orientation found in Study 1.

Libertarians' weaker social interconnectedness is consistent with the idea that they have weaker moral intuitions concerning obligations to and dependence on others (e.g. Moral Foundation Questionnaire scores). If “moral thinking is for social doing” [33], then libertarians lack of social connection naturally means that they have less use for moral thinking. Their distaste for submitting to the needs and desires of others helps explain why libertarians have very different ways of relating to groups, consistent with their lower endorsement of values related to altruism, conformity, and tradition in Study 1, providing convergent evidence for the idea that moral judgment is tightly related to social functioning.

## Conclusions

While not all libertarians endorse the views of Ayn Rand, our findings can be summarized by the three quotations we have presented from her work. We began Study 1 with Rand's exhortation to reject “the morality of altruism,” and we showed that libertarians do indeed reject this morality, as well as all other moralities based on ideas of obligation to other people, groups, traditions, and authorities. Libertarians scored relatively high on just one moral concern: liberty. The libertarian pattern of response was found to be empirically distinct from the responses of liberals and conservatives, both in our cluster analysis of participants and in our principal components analysis of measures. We found strong support for our first prediction: *Libertarians will value liberty more strongly and consistently than liberals or conservatives, at the expense of other moral concerns.*

We introduced Study 2 with Rand's claim that Western culture can only be reborn when it can be founded on “a rational ethics.” Consistent with Rand's writing and psychological research concerning the intuitive origins of moral reasoning [8], we found that libertarians were indeed less emotional (less disgust sensitivity, empathic concern, and neuroticism) than liberals and conservatives. This lack of emotional reactivity may underlie an indifference towards common moral norms, and an attraction to an ideology where these moral codes are absent, libertarianism. The only emotional reaction on which libertarians were not lowest was reactance – the angry reaction to infringements upon one's autonomy – for which libertarians scored higher than both liberals and conservatives. This disposition toward reactance may lead to the moralization of liberty and an attraction to an ideology that exalts liberty above other moral principles – namely, libertarianism.

We also found that libertarians showed a strong preference for and enjoyment of reasoning (higher on utilitarianism, need for cognition, systemizing, and a greater likelihood of answering correctly on the cognitive reflection task). We think it is worth repeating that libertarians were the only one of our three groups for which systemizing scores were higher, in absolute terms, than their empathizing scores, suggesting that libertarians are the only group that may be psychologically prepared for the Randian revolution of “rational ethics.” Thus, we found strong support for our second prediction: *Libertarians will rely upon emotion less – and reason more – than will either liberals or conservatives*.

We introduced Study 3 with Rand's condemnation of love that is not based on a strong sense of self. We found that libertarians do indeed have a strong sense of self and the self's prerogatives, and a correspondingly lower sense of attachment to others. They exhibit a high degree individualism, a low degree collectivism, and generally report feeling less bonding with others, less loving for others, and less feelings of a sense of common identity with others. Libertarians have a lower degree of the broad social connection that typifies liberals as well as a lower degree of the tight social connections that typify conservatives. These social preferences were related to their moral attitudes suggesting that libertarians have less functional use for moral concerns. We found strong support for out third prediction: *Libertarians will be more individualistic and less collectivist compared to both liberals and conservatives.*

### Personality and Ideology

The current research extends past comparisons between liberals and conservatives to a third ideological group — libertarians. Our findings are consistent with the emerging view that personality plays a crucial role in the formation of ideology. As is the case with liberals and conservatives [3], libertarian ideological identification is characterized by specific moral concerns, a level 2 characteristic adaptation in McAdams' [23] model of personality. But why do people develop differential preferences for specific moral concerns? Both McAdams' more general theory and recent theory specifically concerning the development of moral reasoning [8] posit that these constructs are often related to and constrained by level 1 traits; for example, previous research has shown that people who are dispositionally high on openness to experience are more likely to develop liberal values [1], whereas people who are dispositionally high on disgust sensitivity are more likely to develop conservative values [14]. Further, consistent with widely tested theories of motivated reasoning [26], people are likely to moralize their preferences [10], especially their social preferences, given the interplay between social functioning and moral reasoning [30], [33].

The current research not only describes an important ideological group, but also tells a coherent story about how and why some people become libertarians while others become liberals or conservatives. While we cannot establish causality with our correlational data, we can see several cross-level links of the sort described by McAdams and Pals [35] and modeled by Lewis and Bates [9]. People who are dispositionally more (at level 1) open to new experiences and reactant are more likely to find themselves drawn to some classically liberal philosophers (such as John Stuart Mill) and classically liberal values and ideals (such as the superordinate value of individual liberty, at level 2). But if these same people are also highly individualistic and low on empathic concern — if they simply feel the suffering of other people less — then they might feel little emotional attraction to modern liberalism's emphasis on altruism and positive liberty, and turned off by its willingness to compel some citizens to help other citizens (through redistributive tax policies). When they first encounter libertarian philosophy (or read an Ayn Rand novel or hear a Ron Paul speech), they find an ideological narrative (level 3) that resonates with their values and their emerging political likes and dislikes (level 2). They begin identifying themselves as libertarians, which reinforces their moral beliefs. They find it easier to reject statements endorsing altruism (or group loyalty or respect for authority) than they would have before having discovered libertarianism and its rationalist, individualist ethos.

A related way to describe the links between personality and morality is found in Rozin's [10] description of the moralization of preferences. Libertarians' preferences about how to live their lives may have been transformed into a moral value — the value of liberty — in the same way that vegetarians have been found to moralize their eating preferences [78] or non-smokers moralize their aversion to smoke [79]. From a social intuitionist perspective [8], this process is no different from the psychological comfort that liberals attain in moralizing their empathic responses (e.g. [15]) or that social conservatives attain in moralizing their connection to their groups (e.g. [43]). For those who self-identify as libertarian in our sample, their dispositional and motivational profiles all point toward one supreme moral principle: individual liberty.

The current research examined a specific ideological group in the United States, but just as research on other distinctive groups such as patients with brain lesions [30] or psychopaths [80] has been generative for understanding morality more broadly, so too do we hope that the current research is generative for researchers seeking to understand political processes in diverse socio-demographic contexts. The current research, convergent with basic research on the intuitive origins of moral judgment [8], suggests that similar patterns may be found in other groups that favor less government involvement in both social and economic matters, such as the Free Democratic Party of Germany, which advocates reduced economic regulation, greater privacy, and increased rights for homosexuals. Even in countries without a political identity that mirrors American libertarianism, there are likely to be individuals who reject policies driven by empathy for the poor or promotion of tradition, and those individuals may exhibit some of the same dispositional traits that are characteristic of libertarians in the US context, such as a desire for solitude and a preference for rational over emotional experience. However, without the reinforcing characteristics of a narrative that can bring coherence to these dispositions [36], these individuals may not have had adequate opportunity to moralize their preferences [10], and may therefore be more likely to be politically apathetic [81].

### Limitations

This set of studies has two main limitations: our findings rely almost exclusively on self-report measures, and our sample is not representative of the general population. Our reliance on self-report measures is partially mitigated by the fact that we used diverse measures that converge on an extremely consistent picture of libertarianism. The fact that libertarian performance on the Cognitive Reflection Task and their responses to classic moral dilemmas converges with libertarian self-report of their cognitive and emotional style also mitigates some of this concern. Because so little has been written about libertarian psychology, we believe that our very large set of self-report measures is an important first step in characterizing libertarian psychology upon which more methodologically advanced work can build [16]. We hope that this research will inform future researchers who will undoubtedly investigate the relationships we have found using more experimental, behavioral, implicit, and even neuropsychological methods.

Our use of a volunteer internet sample means that we must be cautious in generalizing our findings to the broader population. However, our results generally replicate across gender (see Tables 2, 3, and 4), as well as sub-samples based on the four most common methods of finding our website (via search engines, the New York Times, Edge.org, or by typing in the URL directly – See Table 7), indicating that our findings are robust. Since sub-sample analysis uses implicit browser referrer information that is technically difficult to fake, we can be confident that our results are not the result of any systematic deception by participants. In addition, many of our ancillary findings replicate previous research (e.g. liberals are higher on Openness to Experience [1], and empathy [15], conservatives report higher disgust sensitivity [14]; and greater preference for tradition [4]), which means that our sample likely bears reasonable resemblance to samples used in previous psychological research. Finally, findings based on the yourmorals.org dataset have been successfully replicated in nationally representative U.S. samples (see, for example, Smith & Vaisey, [82], replicating findings about liberal-conservative differences on the Moral Foundations Questionnaire).

**Table 7:** Cohen's d-scores by referrer sub-sample.

		Libertarians vs. Liberals				Libertarians vs. Conservatives		
Scale	Search Engines	NY Times	Edge.Org	Direct URL	Search Engines	NY Times	Edge.Org	Direct URL
Moral Foundations Questionnaire								
Harm	−1.07	−1.18	−1.15	−1.00	−0.52	−0.23	−0.15	−0.27
Fairness	−0.91	−0.99	−0.82	−0.79	−0.08	0.17	0.35	0.18
Ingroup	0.01	0.37	0.34	0.10	−1.19	−0.79	−0.97	−1.14
Authority	−0.05	0.33	0.23	0.04	−1.58	−1.17	−1.33	−1.45
Purity	−0.07	0.17	0.05	−0.11	−1.61	−1.40	−1.65	−1.62
Schwartz Values Scale								
Achievement	−0.17	0.02	0.12	0.21	−0.30	−0.12	0.01	0.04
Benevolence	−0.70	−0.59	−0.67	−0.45	−0.80	−0.46	−0.47	−0.39
Conformity	−0.30	−0.05	−0.18	−0.10	−1.35	−1.11	−1.12	−1.15
Hedonism	0.18	0.07	0.04	0.04	0.46	0.62	0.75	0.48
Power	−0.07	0.17	0.02	0.01	−0.50	−0.19	−0.13	−0.45
Security	−0.23	0.00	−0.23	0.03	−1.12	−0.63	−0.91	−0.74
Self-Direction	0.12	0.23	0.31	0.29	0.44	0.65	0.60	0.74
Stimulation	−0.01	0.09	0.04	−0.02	0.34	0.52	0.33	0.36
Tradition	−0.25	−0.10	−0.32	−0.16	−1.52	−1.06	−1.37	−1.23
Universalism	−0.80	−1.02	−1.10	−0.84	0.19	0.31	0.22	0.26
Big Five Personality Inventory								
Agreeableness	−0.62	−0.46	−0.42	−0.41	−0.54	−0.31	−0.24	−0.37
Conscientiousness	−0.04	−0.20	0.01	−0.10	−0.32	−0.44	−0.06	−0.35
Extraversion	−0.22	−0.18	−0.09	−0.13	−0.26	−0.09	−0.09	−0.19
Neuroticism	−0.13	−0.20	−0.26	−0.20	−0.06	0.03	0.16	0.01
Openness	0.06	−0.01	−0.09	0.02	0.80	0.51	0.37	0.57
Baron-Cohen								
Empathizer	−0.56	−0.77	−0.60	−0.80	−0.47	−0.36	−0.29	−0.43
Systemizer	0.47	0.49	0.54	0.43	0.29	0.24	0.47	0.24
Individualism-Collectivism Scale								
Collectivism - Horizontal	−1.36	−0.95	−0.40	−0.53	−1.03	−0.68	−0.05	−0.52
Collectivism - Vertical	−1.07	−0.22	−0.04	−0.26	−1.98	−0.97	−0.55	−1.05
Individualism - Horizontal	0.29	0.68	0.32	0.86	0.49	0.57	0.32	0.84
Individualism - Vertical	0.23	0.50	0.63	0.77	−0.15	−0.09	−0.02	0.08
Identification with All Humanity Scale								
Identification with Community	−0.28	−0.23	−0.25	−0.36	−0.74	−0.26	−0.53	−0.76
Identification with Country	−0.24	0.20	0.14	−0.14	−0.89	−0.40	−0.85	−1.08
Identification with World	−0.55	−0.86	−0.94	−0.67	0.03	0.01	0.00	0.18

Our sample, while far more diverse than most college samples [83], has specific characteristics that reduce the generalizability of this research. The sample tends to be more politically aware, educated, white, and liberal than a representative U.S. sample would be. This reduces the likelihood of confounds due to race or education, but also means that it remains an open question whether the relationships found would hold within a less educated or more racially diverse group. In addition, political and moral differences are likely more salient in the context of our website, meaning that effect sizes may be increased in this setting. The mean values for libertarians in our sample are likely quite different than the mean values for these measures if we were able to examine the population as a whole. However, whereas the mean values derived from our dataset may differ from national averages, the relationships between variables in our dataset have been found to be comparable to nationally representative samples [84].

Our use of a volunteer internet sample gave us at least three benefits in terms of data quality. First, because volunteers are often more educated and motivated, such samples often show less random measurement error, less survey satisficing, and less social desirability bias compared to nationally representative samples [85]–[87]. Second, unlike many surveys conducted by telephone, we were able to use full and well-validated scales to measure each construct, rather than relying on just one or two items. And third, because nationally representative samples are expensive to procure, they rarely involve more than 2,000 respondents. If self-described libertarians comprise less than 10% of the U.S. population, then nationally representative samples rarely include enough libertarians to make the sort of comparisons we were able to make using our much larger dataset.

While our sample represents a large number of libertarians, it may or may not represent the majority. Not withstanding our cluster analysis in Study 1, libertarianism may also be studied as a dimension that an individual may endorse to varying degrees rather than as a discrete kind of person, which may be one of the reasons that national surveys typically do not measure identification as libertarian. Self-identification as ‘libertarian’ can change meaning over time, further complicating the issue. William James [88] felt that he could best study the human experience of religion by studying its extreme forms. Our sample may be taken from one end of the libertarian dimension, specifically those who are willing to take the psychological step of self-identifying specifically as libertarian. Libertarianism may be a dimension that may exist in both liberals and conservatives to varying degrees, as both liberals and conservatives endorse liberty as a moral value in different domains. In learning about this group of individuals, perhaps we can learn something about the forces that push all individuals towards or away from endorsing liberty as a moral end.

### Summary

Political and social psychologists often study ideology on a unidimensional liberal-conservative spectrum, but the real world is clearly more complex. As psychologists advance in studying the personality traits associated with liberalism and conservatism, our findings confirm the value of this approach and extend its reach by describing a heretofore-neglected yet politically important group – libertarians. Libertarians have a unique moral-psychological profile, endorsing the principle of liberty as an end and devaluing many of the moral concerns typically endorsed by liberals or conservatives. Although causal conclusions remain beyond our current reach, our findings indicate a robust relationship between libertarian morality, a dispositional lack of emotionality, and a preference for weaker, less-binding social relationships. These findings are consistent with previous research on the dispositional origins of moral judgment. By focusing on one understudied ideological group, the findings provide further evidence concerning the closely intertwined nature of personality, values, and political ideology.

## Supporting Information

Appendix S1 **Liberty Items.**
(DOC)
