# Supplementary material for: Understanding Libertarian Morality: The Psychological Dispositions of Self-Identified Libertarians
Source: PLoS One. 2012 Aug 21;7(8):e42366. doi: 10.1371/journal.pone.0042366 (PMC3424229; doi:10.1371/journal.pone.0042366)
Supplement: Appendix S1 — Liberty Items. (DOC) [file pone.0042366.s001.doc]

Appendix A – Liberty Items

Economic/Government Liberty:

Whether or not private property was respected (relevance rating)

People who are successful in business have a right to enjoy their wealth as they see fit

Society works best when it lets individuals take responsibility for their own lives without telling them what to do.

The government interferes far too much in our everyday lives.

The government should do more to advance the common good, even if that means limiting the freedom and choices of individuals. (Reverse scored)

Property owners should be allowed to develop their land or build their homes in any way they choose, as long as they don't endanger their neighbors.

Lifestyle Liberty:

Whether or not everyone was free to do as they wanted. (relevance rating)

I think everyone should be free to do as they choose, so long as they don't infringe upon the equal freedom of others.

People should be free to decide what group norms or traditions they themselves want to follow.
